# Supplementary material for: Bacterial cell surface nanoenvironment requires a specialized chaperone to activate a peptidoglycan biosynthetic enzyme
Source: bioRxiv. 2023 Oct 6:2023.10.06.561273. Preprint. [Version 1] doi: 10.1101/2023.10.06.561273 (PMC10659427; doi:10.1101/2023.10.06.561273)
Supplement: 1 [file NIHPP2023.10.06.561273V1-supplement-1.pdf]

bioRxiv preprint doi: https://doi.org/10.1101/2023.10.06.561273; this version posted October 6, 2023. The copyright holder for this preprint (which was not certified by peer review) is the author/funder. This article is a US Government work. It is not subject to copyright under 17 USC 105 and is also made available for use under a CC0 license.

SUPPORTING INFORMATION

Table S1. Cortex peptidoglycan analysis of WT and Δ*spoVK* strains. Extracted peptidoglycan from developing forespores of the wild type and Δ*spoVK* strains harvested 5 h after the onset of sporulation was digested with mutanolysin and separated using HPLC. Muropeptides were identified based on previous studies of elution times[1,2]. Peaks were then integrated, and peptidoglycan structural parameters were calculated. Errors are S.D.

PG species	WT	Δ*spoVK*
% muramic acid as lactam	33.3 ± 1.5	6.5 ± 3.0
% muramic acid with alanine	20.6 ± 0.6	19.5 ± 0.9
% muramic acid with tripeptide	15.9 ±1.9	42.9 ± 3.7
% muramic acid with tetrapeptide	30.2 ± 0.4	31.2 ± 1.4
% muramic acid with pentapeptide	0.0 ± 0.0	0.0 ± 0.0
% peptide as tetrapeptide	65.6 ± 3.0	42.2 ± 2.5
% peptide as tripeptide	34.4 ± 3.0	57.8 ± 2.5
% peptide in crosslinks	19.0 ± 0.6	20.4 ± 2.2
% peptide in effective crosslinks	19.0 ± 0.6	20.4 ± 2.2
% disaccharide units in disaccharides	36.4 ± 3.0	87.1 ± 6.0
% disaccharide units in tetrasaccharides	54.7 ± 2.8	12.9 ± 6.0
% disaccharide units in hexasaccharides	9.0 ± 0.5	0.0 ± 0.0
% lactam in regular distribution	82.1 0.9	100.0 ± 0.0
% disaccharide peptide crosslinked	23.8 ± 0.3	20.4 ± 2.2
% tetrasaccharide peptide crosslinked	5.0 ± 0.2	0.0 ± 0.0
% lactam reduced	0.2 ± 0.0	0.2 ± 0.0
% tripeptide crosslinked	39.6 ± 0.8	31.1 ± 1.7
% tetrapeptide crosslinked	8.2 ± 0.5	5.7 ± 4.7
% disaccharide per effective crosslink	11.5 ± 0.7	6.6 ± 0.4
% muramic acid with crosslink	8.8 ± 0.5	15.1 ± 0.9

[1]DOI: 10.1128/JB.182.16.4491-4499.2000
[2]DOI: 10.1128/jb.178.22.6451-6458.1996

bioRxiv preprint doi: https://doi.org/10.1101/2023.10.06.561273; this version posted October 6, 2023. The copyright holder for this preprint (which was not certified by peer review) is the author/funder. This article is a US Government work. It is not subject to copyright under 17 USC 105 and is also made available for use under a CC0 license.

Table S2. Bacterial Strains.

Bacillus subtilis strains used in this study		
Strain	Genotype or description	Reference
PY79	Prototrophic derivative of *B. subtilis* 168	Youngman et al., 1984
KP73	Δ*spoIVA::neo*	Price and Losick, 1999
KR160	*thrC::gfp-spoIVA spec*	Ramamurthi and Losick, 2008
TD549	Δ*spoVK::erm thrC::gfp-spoIVA spec*	This study
TD520	Δ*spoVK::erm*	This study
TD513	Δ*spoVK::erm amyE::spoVK cat*	This study
TD514	Δ*spoVK::erm amyE::spoVK*^{A5V} *cat*	This study
JPC221	Δ*spoIVA::neo thrC::spoIVA*^{T70A,T71A} *spec*	Castaing et al., 2013
TD524	Δ*spoIVA::neo thrC:: spoIVA*^{T70A,T71A} *spec* Δ*spoVK::erm*	This study
TD530	Δ*spoIVA::neo thrC:: spoIVA*^{T70A,T71A} *spec* Δ*spoVK::erm amyE::spoVK cat*	This study
TD531	Δ*spoIVA::neo thrC:: spoIVA*^{T70A,T71A} *spec* Δ*spoVK::erm amyE::spoVK*^{A5V} *cat*	This study
TD523	Δ*spoIVA::neo thrC::spoIVA*^{D97A} *spec* Δ*spoVK::erm*	This study
TD528	Δ*spoIVA::neo thrC::spoIVA*^{D97A} *spec* Δ*spoVK::erm amyE::spoVK cat*	This study
TD529	Δ*spoIVA::neo thrC::spoIVA*^{D97A} *spec* Δ*spoVK::erm amyE::spoVK*^{A5V} *cat*	This study
KR438	Δ*spoIVA::neo thrC::spoIVA*^{K30E} *spec*	Ramamurthi and Losick, 2008
TD817	Δ*spoIVA::neo thrC::spoIVA*^{K30E} *spec* Δ*spoVK::erm*	This study
TD818	Δ*spoIVA::neo thrC::spoIVA*^{K30E} *spec* Δ*spoVK::erm amyE::spoVK cat*	This study
TD819	Δ*spoIVA::neo thrC::spoIVA*^{K30E} *spec* Δ*spoVK::erm amyE::spoVK*^{A5V} *cat*	This study
TD845	Δ*spoIVA::neo amyE::spoIVA*^{D97A} *cat thrC::GFP-spoIVA*^{D97A} *spec* Δ*spoVK::erm pyrD::spoVK cat::tet*	This study
TD846	Δ*spoIVA::neo amyE::spoIVA*^{T70A,T71A} *cat thrC::GFP-spoIVA*^{T70A,T71A} *spec* Δ*spoVK::erm pyrD::spoVK cat::tet*	This study
TD854	Δ*spoIVA::neo amyE::spoIVA*^{K30E} *cat thrC::GFP-spoIVA*^{K30E} *spec* Δ*spoVK::erm pyrD::spoVK cat::tet*	This study
TD848	Δ*spoIVA::neo amyE::spoIVA*^{D97A} *cat thrC::GFP-spoIVA*^{D97A} *spec* Δ*spoVK::erm pyrD::spoVK*^{A5V} *cat::tet*	This study
TD849	Δ*spoIVA::neo amyE::spoIVA*^{T70A,T71A} *cat thrC::GFP-spoIVA*^{T70A,T71A} *spec* Δ*spoVK::erm pyrD::spoVK*^{A5V} *cat::tet*	This study
TD855	Δ*spoIVA::neo amyE::spoIVA*^{K30E} *cat thrC::GFP-spoIVA*^{K30E} *spec* Δ*spoVK::erm pyrD::spoVK*^{A5V} *cat::tet*	This study
JB103	Δ*spoIVA::neo thrC::spoIVA*^{D97A} *spec*	This study
TD604	*amyE::spoVK-linker-GFP cat*	This study
TD652	Δ*spoIVA::neo amyE::spoVK-linker-GFP cat*	This study
TD675	Δ*spoVK::erm amyE::spoVK cat pyrD::spoVK-linker-GFP cat::tet*	This study
TD682	Δ*spoIVA::neo thrC::spoIVA*^{D97A} Δ*spoVK::erm amyE::spoVK cat pyrD::spoVK-linker-GFP cat::tet*	This study
TD684	Δ*spoIVA::neo thrC::spoIVA*^{T70A,T71A} Δ*spoVK::erm amyE::spoVK cat pyrD::spoVK-linker-GFP cat::tet*	This study

bioRxiv preprint doi: https://doi.org/10.1101/2023.10.06.561273; this version posted October 6, 2023. The copyright holder for this preprint (which was not certified by peer review) is the author/funder. This article is a US Government work. It is not subject to copyright under 17 USC 105 and is also made available for use under a CC0 license.

TD836	*ΔspoIVA::neo thrC::*$spoIVA^{K30E}$ *ΔspoVK::erm amyE::spoVK cat pyrD::spoVK-linker-GFP cat::tet*	This study
TD557	*ΔspoIVA::neo thrC::*$spoIVA^{D97A}$ *spec amyE::spoVK cat*	This study
TD558	*ΔspoIVA::neo thrC::*$spoIVA^{D97A}$ *spec amyE::*$spoVK^{A5V}$ *cat*	This study
TD563	*ΔspoIVA::neo thrC::*$spoIVA^{T70A,T71A}$ *spec amyE::spoVK cat*	This study
TD564	*ΔspoIVA::neo thrC::*$spoIVA^{T70A,T71A}$ *spec amyE::* $spoVK^{A5V}$ *cat*	This study
TD859	*ΔspoIVA::neo thrC::*$spoIVA^{K30E}$ *spec amyE::spoVK cat*	This study
TD860	*ΔspoIVA::neo thrC::*$spoIVA^{K30E}$ *spec amyE::* $spoVK^{A5V}$ *cat*	This study
TD574	*ΔspoVK::erm amyE::*$spoVK^{\Delta 2\text{-}42}$ *cat*	This study
TD575	*ΔspoVK::erm amyE::*$spoVK^{K105A,T106A}$ *cat*	This study
TD576	*ΔspoVK::erm amyE::*$spoVK^{D162A,E163A}$ *cat*	This study
TD578	*ΔspoVK::erm amyE::*$spoVK^{R218A,R276A}$ *cat*	This study
TD597	*amyE::spoVK cat*	This study
TD598	*amyE::*$spoVK^{\Delta 2\text{-}42}$ *cat*	This study
TD599	*amyE::*$spoVK^{K105A,T106A}$ *cat*	This study
TD600	*amyE::*$spoVK^{D162A,E163A}$ *cat*	This study
TD602	*amyE::*$spoVK^{R218A,R276A}$ *cat*	This study
TD883	*amyE::*P_{spoVID}*-*$spoVK^{D162A,E163A}$*-*His_6 *cat*	This study
TD884	*amyE::*P_{spoVID}*-*$spoVK^{D162A,E163A}$*-FLAG cat*	This study
TD1193	*amyE::*P_{spoVID}*-*$spoVK^{A5V,D162A,E163A}$*-FLAG cat*	This study
TD651	*ΔspoVID::neo amyE::spoVK-linker-GFP cat*	This study
TD695	*ΔspoVID::neo amyE::*$spoVID^{T532G}$ *cat pyrD::spoVK-linker-GFP cat::tet*	This study
TD1257	*ΔspoIVA::erm ΔspoVID::neo thrC::spoIVA spec amyE::*$spoVID^{501\text{-}575}$ *pyrD::spoVK-linker-GFP cat::tet*	This study
KR394	*ΔspoIVA::neo thrC::spoIVA spec*	Ramamurthi et al., 2006
JB171	*ΔspoIVA::erm ΔspoVID::neo thrC::spoIVA spec*	This study
JB174	*ΔspoIVA::erm ΔspoVID::neo thrC::spoIVA spec amyE::spoVID cat*	This study
TD892	*ΔspoIVA::erm ΔspoVID::neo thrC::spoIVA spec amyE::*$spoVID^{501\text{-}575}$	This study
TD893	*ΔspoIVA::erm ΔspoVID::neo thrC::spoIVA spec amyE::*$spoVID^{526\text{-}575}$	This study
TD1268	*ΔspoIVA::erm ΔspoVID::neo thrC::spoIVA spec amyE::*P_{spoVID}*-*$spoVK^{D162A\text{-}E163A}$*-FLAG cat*	This study
TD1281	*ΔspoIVA::erm ΔspoVID::neo thrC::spoIVA spec amyE::* P_{spoVID}*-*$spoVK^{D162A\text{-}E163A}$*-FLAG cat sacA::GFP-*$spoVID^{501\text{-}575}$ *cat::tet*	This study
TD1267	*ΔspoIVA::neo thrC::spoIVA spec amyE::* P_{spoVID}*-*$spoVK^{D162A\text{-}E163A}$*-FLAG cat*	This study
TD1277	*ΔspoIVA::neo thrC::spoIVA spec amyE::* P_{spoVID}*-*$spoVK^{D162A\text{-}E163A,\Delta 2\text{-}6}$*-FLAG cat*	This study
TD1283	*ΔspoIVA::neo thrC::spoIVA spec amyE::* P_{spoVID}*-*$spoVK^{D162A\text{-}E163A,A5V}$*-FLAG cat*	This study
TD1278	*ΔspoIVA::erm ΔspoVID::neo thrC::spoIVA spec amyE::*P_{spoVID}*-*$spoVK^{D162A\text{-}E163A,\Delta 2\text{-}6}$*-FLAG cat*	This study
TD1284	*ΔspoIVA::erm ΔspoVID::neo thrC::spoIVA spec amyE::* P_{spoVID}*-*$spoVK^{D162A\text{-}E163A,A5V}$*-FLAG cat*	This study
SC765	*amyE::*P_{spoVM}*-SpoVM-Iphluorin-cat*	This study
SC766	*amyE::*P_{spoIIQ}*-Iphluorin-cat*	This study
SC767	*amyE::*P_{spoVM}*-Iphluorin-cat*	This study
SC777	*thrC::*$P_{hyperspank}$*-Iphluorin-erm*	This study

bioRxiv preprint doi: https://doi.org/10.1101/2023.10.06.561273; this version posted October 6, 2023. The copyright holder for this preprint (which was not certified by peer review) is the author/funder. This article is a US Government work. It is not subject to copyright under 17 USC 105 and is also made available for use under a CC0 license.

Escherichia coli strains used in this study		
Strain	Genotype and description	Reference
BL21(DE3)	*pTD211* (*pET28a* backbone, P_{T7}-*spoVK-His*$_6$)	This study
BL21(DE3)	*pTD308* (*pET28a* backbone, P_{T7}- *spoVK*^{D162A,E163A}-*His*$_6$)	This study

bioRxiv preprint doi: https://doi.org/10.1101/2023.10.06.561273; this version posted October 6, 2023. The copyright holder for this preprint (which was not certified by peer review) is the author/funder. This article is a US Government work. It is not subject to copyright under 17 USC 105 and is also made available for use under a CC0 license.

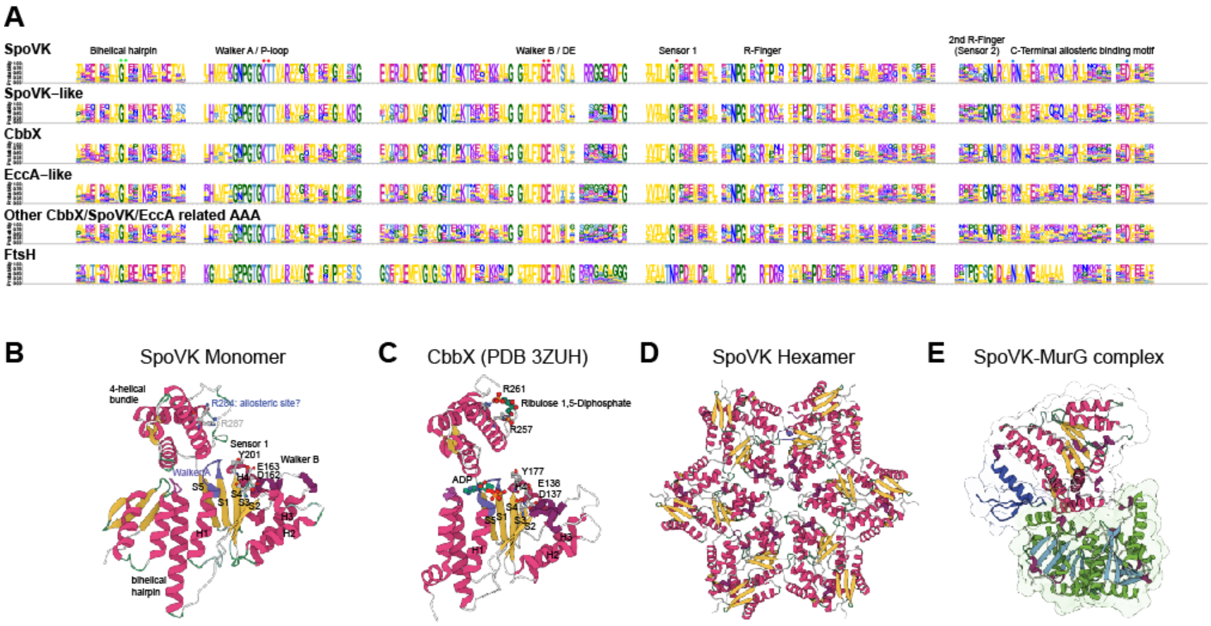

Figure S1. Sequence and structural comparison between SpoVK and members of the SpoVK-EccA-like AAA+ ATPase clade. (A) Sequence logo displaying conservation of amino acid residues in the SpoVK-EccA-like AAA+ ATPase clade. Letters represent amino acid abbreviations; height of each letter represents the probability of conservation among orthologs of the family. The first and second arginine fingers are marked with red dots. Blue dots are conserved residues contacting the allosteric ligand ribulose bisphosphate. (B-E) Cartoon depictions of (B) AlphaFold2 prediction of SpoVK structure, (C) CbbX from *Cereibacter sphaeroides* (PDB 3ZUH), (D) AlphaFold-Multimer prediction of SpoVK hexamer, and (E) AlphaFold-Multimer prediction of SpoVK-MurG complex.

bioRxiv preprint doi: https://doi.org/10.1101/2023.10.06.561273; this version posted October 6, 2023. The copyright holder for this preprint (which was not certified by peer review) is the author/funder. This article is a US Government work. It is not subject to copyright under 17 USC 105 and is also made available for use under a CC0 license.

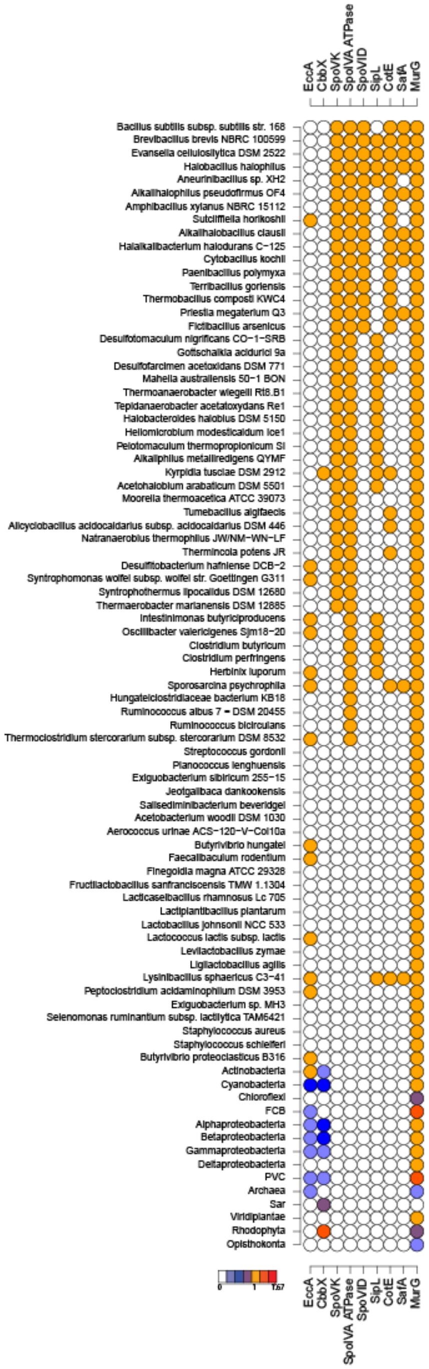

Figure S2. Phyletic pattern vectors of EccA, CbbX, SpoVK, SpoIVA ATPase, SpoVID, SipL, CotE, SafA, and MurG domain proteins. The presence or absence of the protein in the clade or organism (Firmicutes) are shown. The legend shows the color gradient for normalized counts that were scaled by square root.

bioRxiv preprint doi: https://doi.org/10.1101/2023.10.06.561273; this version posted October 6, 2023. The copyright holder for this preprint (which was not certified by peer review) is the author/funder. This article is a US Government work. It is not subject to copyright under 17 USC 105 and is also made available for use under a CC0 license.

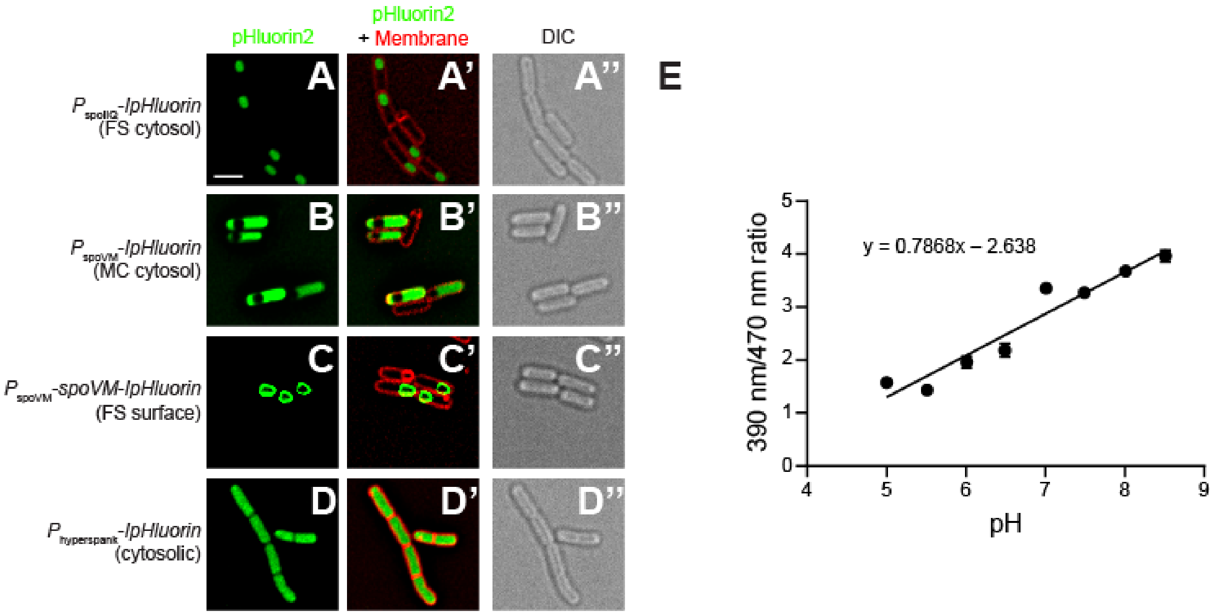

Figure S3. Subcellular localization of various IpHluorin constructs during sporulation. Fluorescence micrographs of *B. subtilis* (A-C'') at t = 4 h after induction of sporulation producing (A-A'') IpHluorin in the forespore, expressed under control of the *spoIIQ* promoter, (B-B'') IpHluorin in the mother cell, expressed under control of the *spoVM* promoter, or (C-C'') SpoVM-IpHluorin at the forespore surface, expressed in the mother cell under control of the *spoVM* promoter; or (D-D'') during vegetative growth producing IpHluorin, expressed under control of an IPTG-inducible promoter at t = 2 h after IPTG induction. (A-D) fluorescence from IpHluorin; (A'-D') overlay, IpHluorin and membranes visualized using FM4-64; (A''-D'') differential interference contrast. Strains: SC765, SC766, SC767, and SC777. Scale bar: 2 µm. (E) Calibration curve of ratio of fluorescence emission at 510 nm when excited at either 390 nm or 470 nm ratio as a function of media pH.
